# Ovary stereological features and serum biochemical factors following induction of polycystic ovary syndrome with testosterone enanthate in mice: An experimental study

**Published:** 2018-04

**Authors:** Zahra Kalhori, Malek Soleimani Mehranjani, Mehri Azadbakht, Mohammad Ali Shariaatzadeh

**Affiliations:** 1 *Department of Biology, Faculty of Science, Arak University, Arak, Iran.*; 2 *Department of Biology, Faculty of Science, Razi University, Kermanshah, Iran. *

**Keywords:** Polycystic ovary syndrome, Inflammation, Oxidative stress, Mice

## Abstract

**Background:**

Polycystic ovary syndrome (PCOS) is an endocrine disorder featured by insulin resistance and hyperandrogenism. Testosterone enanthate can induce PCOS in mice models.

**Objective:**

We investigated the ovary stereological features along with the oxidative stress and inflammatory factors in mice following PCOS induction using testosterone enanthate.

**Materials and Methods:**

Twelve female NMRI mice (3 wk old) were divided into 2 groups (n=6/each): Control and PCOS. PCOS was induced through daily injections of testosterone enanthate (1 mg/100g subcutaneous s.c for 5 wk). Finally, ovaries were studied stereologically. The serum levels of the follicle-stimulating hormone, luteinizing hormone, testosterone, interleukin-6, and tumor necrosis factor-α were measured using ELISA kit. Serum levels of Malondialdehyde and the antioxidant capacity were measured relatively using thiobarbituric acid and ferric reducing antioxidant power assay.

**Results:**

The mean total volume of ovary and the mean volume of cortex (p<0.001), volume of oocyte in the preantral (p=0.011) and antral follicle (p=0.015), thickness of zona pellucida (p=0.016), the number of antral follicles (p=0.012), the serum levels of follicle-stimulating hormone (p<0.001) and the antioxidant capacity (p=0.020) reduced significantly in the PCOS group compared to the control. The number of primary (p=0.017) and preantral (p=0.006) follicles and the serum levels of testosterone (p<0.001), Luteinizing hormone (p=0.002), Malondialdehyde, Interleukin 6 and Tumor necrosis factor-α (p<0.001) showed a significant increase in the PCOS group compared to the control.

**Conclusion:**

Testosterone enanthate induced PCOS causes stereological features in the ovary, increases the oxidative stress and inflammatory markers in mice.

## Introduction

Polycystic ovary syndrome (PCOS) is the most common endocrine disorder that occurs in women at the reproductive age and is characterized by hyperandrogenism, chronic anovulation, polycystic ovaries, hyperinsulinemia and the accumulation of small subcortical follicles (1, 2). The intra-ovarian hyperandrogenism may be the main culprit for follicular excess, anovulation, and atresia of follicles in polycystic ovaries (3, 4). Although the etiology of PCOS is still not determined, studies suggest that oxidative stress and inflammation may play an important role in disturbing of metabolic and reproductive found in PCOS (5-7). Elevated lymphocytes and macrophages in PCOS ovaries could induce follicular atresia by various cytokines acting on the theca and granular cells, consequently, dominant follicles cannot be generated (7). Another common feature of PCOS is the reproductive hormone dysregulation involving increased plasma level of Luteinizing hormone (LH) which has been associated with a significant decrease in fertilization rate, oocyte maturation and impaired embryo quality which consequently leads to impaired pregnancy rates (8, 9). Understanding the pathogenesis of PCOS could help to make a more effective treatment therefore to study PCOS, animal models are required that have similarities to human (10). In this case exposure of sheep and primates to testosterone has developed models that show similarities to PCOS women such as polycystic ovaries, anovulation, insulin resistance and LH hypersecretion but both models have long developmental periods and are expensive whereas mouse models have a short reproductive lifespan and genetic manipulations are feasible (11-14). Testosterone enanthate is an anabolic steroid with androgenic properties which has been considered as a PCOS inducer (15), although oxidative stress and inflammation are the two main pathways involved in the pathogenesis of PCOS, they have been less investigated in mice models with testosterone enanthate induced PCOS. 

Therefore we aimed to evaluate the histological changes of the ovaries using stereological techniques along with the assessment of the biochemical factors, inflammatory and oxidative stress markers in mice following PCOS induction by testosterone enanthate.

## Materials and methods

**Animals and polycystic ovary syndrome induction**

Twelve immature female NMRI mice (3 wk old) were purchased from Pasteur Institute and kept in Arak University animal house under standard conditions (22±2^o^C and 12 hr light/dark cycles) and divided into 2 groups (n=6/each): PCOS group which were injected daily with testosterone enanthate (1 mg/100 g body weight dissolved in sesame oil) (4) and the control group which were injected only with vehicle. Treatment was carried out for 5 wks and at the end of the treatment, mice were sacrificed and their ovaries and serum were collected for stereological and biochemical factor analysis, respectively. 

**Stereological study**

The excised ovaries from the PCOS and control groups were immediately fixed in Bouin’s solution. After 24 hr fixation, ovaries were dehydrated in ascending series of ethanol, cleared with xylene and embedded in paraffin wax. Isotropic uniform random sections were obtained using the ‘isector’ method (16). In this method, the filled spherical modules with paraffin used to insert each ovary. Then modules were rotated in a random manner and 5 and 20 μm thick sections were prepared using microtome. Sections were stained with haematoxylin and eosin and 12 sections per animal were examined stereologically.

**Estimating the total volume of the ovary, the volume of cortex and medulla**

Using Cavalieri method the total volume of the ovary and the volume of cortex and medulla was estimated (16). Twelve sections from 5-µm thick sections were transferred to the working table using the micro-projector with 4× magnification. The resulting points from the randomly superimposed probe on the images were then counted and the total volume of the ovary was estimated using the following formula:

$V_{\mathrm{total ovary}}=\sum_{i=1}^{n}p\times a(p)\times t$

Where $\sum_{i=1}^{n}P$ is the total number of points superimposed on the image, (t) is the thickness of the section and a (p) is the area associated with each point. The volume density of each ovary compartment was calculated as follows:

$V_{v_{\mathrm{cortex}}}=\frac{\sum_{i=1}^{n}P_{\mathrm{cortex}}}{\sum_{i=1}^{n}P_{\mathrm{total}}}$

Where$\sum_{i=1}^{n}P_{\mathrm{total}}$ the total number of is counted points and$\sum_{i=1}^{n}P_{\mathrm{cortex}}$ is the total number of superimposed points on the cortex. In turn, the volume density (Vv) was multiplied by the total volume of the ovary to estimate the volume of cortex and medulla (16).

**Estimating the number of follicles**

In order to estimate the number of follicles, the optical dissector method was used. Using systematic random sampling 12 sections were selected out of 20-µm thick sections and were studied by a microscope with 100x magnification. Using a microcator connected to a computer and microscope, the movement of the stage in the z-axis was measured. In order to avoid the cutting artifacts, 5 µm from the bottom and top of the sections were ignored as a guard area. Any nucleus that lied in the frame and having no contact with lines of the frame was selected. Different types of follicles were identified based on the Myers and colleagues (17). The number density (Nv) of different types of follicles was estimated as:

$\mathrm{NV}=\frac{\sum_{i-1}^{n}Q}{h\times \sum_{n-1}^{n}P\times a/f}$

Σ*Q *is the total number of the counted follicles in the dissector height (h), a/f is the frame area in the true tissue scale and Σ*P* is the total number of the points superimposed. The total number of the follicles was estimated by multiplying the numerical density (Nv) by the total volume of the ovary, (N_total_=N_V_×V_total_) (16).

**Estimation of the volume of oocyte and its nuclei**

The oocytes volume and their nuclei were estimated using the nucleator method. An average of 12 sections from 20-µm thick sections was randomly selected. Then, the selected follicles were studied using the microscope with a 100× magnification. In order to estimate the volume of the oocyte, the distance from the center of the nucleolus to the oocyte membrane was measured and to estimate the volume of the oocyte nucleus, the distance from the center of the nucleolus to the nucleus membrane was measured (17-18). The mean volume was estimated through:

$V_{n}=4/3\pi \times \bar{L_{n}^{3}}$

Ln is the distance from the sampling point to the edge of the particle (such as the nucleus or the oocyte).

**Zona pellucida (ZP) thickness**

To calculate the mean ZP thickness, an average of 12 sections was randomly selected among 5μm thick sections. In order to determine measurement sites, the specific line grid (three parallel lines) was randomly superimposed on the sampled fields. The orthogonal intercept method was used to calculate the mean thickness of the ZP. In this method, the length of a perpendicular line extended from the inner membrane to the outer surface of zona pellucida at each intercept of the line of the grid with zona membrane considered as orthogonal intercept. An average of 110 measurements was estimated and the harmonic mean thickness was calculated using the following formula (19):

Harmonic mean thickness=8π /3×Harmonic mean of orthogonal intercepts, where the harmonic mean= number of measurements/ sum of the reciprocal of orthogonal intercepts lengths= number of measurements/ $\left(\frac{1}{{\mathrm{oi}}_{1}}+\frac{1}{{\mathrm{oi}}_{2}}+\frac{1}{{\mathrm{oi}}_{3}}+\frac{1}{{\mathrm{oi}}_{4}}+\ldots \right)$

**Hormones assay**

After 35 days, blood samples were collected and centrifuged (3000 g, 5 min) and serum was analyzed in duplicates with the FSH kit (Cat No. MBS260957, San Diego, CA) with a sensitivity of 0.5 mIU/ml, LH kit (Cat. No: MBS041300, San Diego, CA) with a sensitivity of 0.1 mIU/ml and the testosterone kit (cat. NO: DKO002, Italy) with a sensitivity of 0.07ng/ml, according to the manufacturer’s instructions.

**Inflammatory cytokine assay**

Serum tumor necrosis factor-α (TNF-α) and Interleukin 6 (IL-6) were measured by the ELISA kits (TNF-α cat NO: BMS607/3, eBioscience, USA) with a sensitivity of 2.97 pg/ml and (IL-6 cat NO: BMS603/2, eBioscience, USA) according to the manufacturer’s instructions.

**Evaluating oxidative stress parameters**

Malondialdehyde (MDA) concentration was determined by the method described by Buege and Aust based on thiobarbituric acid reactivity (20). Briefly, 1 mL of Trichloroacetic acid TCA-2-thiobarbituric acid (TBA)-HCl reagent was added to 50µl of serum. Then samples are kept in boiling water for 10 min. After cooling, the reaction mixture was centrifuged, then supernatant was taken and absorbance of the pink color formed was read at 535 nm and the concentration of MDA was calculated using 1.56×10^5^ mol^-1^ cm^-1^ as molar absorbance coefficient. Total antioxidant capacity was measured as FRAP by UV spectrophotometry (21). Briefly, 50µl of serum was added to FRAP reagent (10 parts of 300 mM sodium acetate buffer at pH 3.6, 1 part of 10.0 mM TPTZ solution and 1 part of 20.0 mM FeCl_3_. 6H_2_O solution) and the reaction mixture is incubated at 37^o^C for 10 min and the absorbance was measured at 593 nm.

**Vaginal smear**

To assess the stages of the estrous cycle, vaginal smears were taken once each day. 50 microliters of saline were inserted into the vagina and then placed on a slide. Vaginal smears were stained using the Papanicolaou stain and studied using the light microscope (10× magnification) (22).

**Ethical consideration**

All animal procedures were approved by the Ethics Committee of Arak medical Science University (IR.ARAKMU.REC.1395.79).

**Statistical analysis**

The results were statistically analyzed using SPSS (Statistical Package for the Social Sciences, version 16.0, SPSS Inc, Chicago, Illinois, USA) by Student’s *t*-test except the concentration of TNF-α and IL-6 which were analyzed by Mann-Whitney U-test and the means were considered significantly different at p<0.05.

## Results

**Ovarian histological aspects**

**Figure 1:**
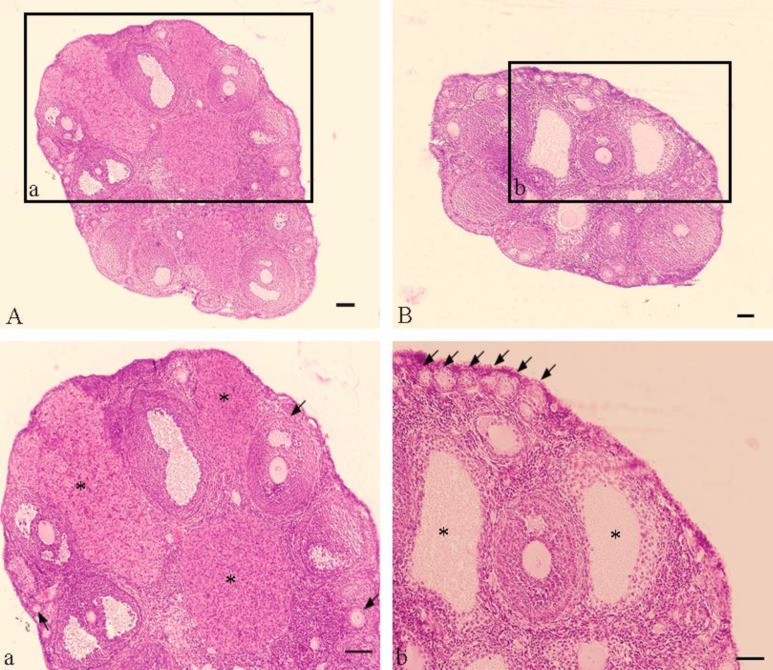
Histological sections of ovaries in PCOS and control groups (5-µm-thick sections with hematoxylin and eosin (H&E) staining). (A) Control group. (a) Magnification of the solid line square in A: indicating follicles at different stages of development (arrows) and the presence of the corpora lutea (stars). (B) PCOS group. (b) Magnification of the solid line square in B: indicating primary follicles (arrows) and cystic follicles (stars). Scale bar= 100 µm

The size of the ovaries reduced in the PCOS group compared to the control group. Microscopic observations of the ovaries in the PCOS group revealed the absence of corpus luteum (anovulation), the presence of cystic follicles and an increased number of primary and preantral follicles (Figure 1).

**The volume of ovary, cortex and medulla**

The mean total volume of the ovary and the mean volume of cortex decreased significantly in the PCOS group compared to the control group (p<0.001). The mean volume of medulla showed no significant difference in any of the groups (p=0.20) (Table I).

**Number of follicles**

The mean number of the primary (p=0.017) and preantral (p=0.006) follicles increased significantly in the PCOS group compared to the control group, while the mean number of the antral follicles was significantly lower in the PCOS group compared to the control group (p=0.012). The mean number of the primordial follicles showed no significant difference in any of the groups (p=0.45) (Table II).

**The volume of oocytes and their nuclei and thickness of zona pellucida**

The mean volumes of the oocytes in the pre-antral (p=0.011) and antral (p=0.015) follicles and the thickness of zona pellucida in the pre-antral (p=0.016) and antral (p=0.011) follicles were significantly lower in the PCOS group compared to the control group (Table III, IV). The mean volumes of the oocytes in the primordial (p=0.35) and primary (p=0.90) follicles and the volumes of oocytes nuclei in the primordial, primary, pre-antral and antral follicles showed no significant difference in any of the groups (Table III).

**Hormone assay**

The level of testosterone (p<0.001) and LH (p=0.002) hormones were significantly higher in the PCOS group when compared to the control group, while the FSH concentration was significantly lower in the PCOS group when compared to the control group (p<0.001) (Table V).

**Oxidative stress and inflammatory markers**

There was a significant increase in the concentration of MDA, IL-6, and TNF-α (p<0.001) and a significant decrease in the total antioxidant capacity in the PCOS group in comparison to the control group (p=0.020) (Table VI).

**Vaginal cytology**

Vaginal cytologic examination revealed that PCOS mice stopped in the diestrus phase. While control mice exhibited regular estrous cycles.

**Table I:** Comparison of the mean total volume of the ovary, the mean volume of cortex and medulla (mm^3^) in different groups of mice 5 wk after treatment with testosterone enanthate

**Groups**	**Volume of ovary**	**Volume of cortex**	**Volume of medulla**
Control	2.00 ± 0.10^a^	1.82 ± 0.08^a^	0.18 ± 0.01^a^
PCOS	1.51 ± 0.09^b^	1.34 ± 0.09^b^	0.17 ± 0.02^a^
p-value	0.001	0.001	0.20

Values are means ± SD. a and b letters in each column indicate a significant difference between the groups (Student’s *t* test).

PCOS: Polycystic ovary syndrome

**Table II.** Comparison of the mean number of primordial, primary, pre-antral, and antral follicles in different groups of mice 5 wk after treatment with testosterone enanthate

**Groups**	**Primordial follicles**	**Primary follicles**	**Preantral follicles**	**Antral follicles**
Control	1728.54 ± 78.63^a^	509.60 ± 26.15^a^	325.71 ± 16.12^a^	122.78 ± 10.45^a^
PCOS	1775.92 ± 69.84^a^	651.04 ± 41.08^b^	506.96 ± 43.30^b^	84.79 ± 9.34^b^
p-value	0.45	0.017	0.006	0.012

Values are means ± SD. a and b letters in each column indicate a significant difference between the groups (Student’s *t* test).

PCOS: Polycystic ovary syndrome

**Table III:** Comparison of the mean volume of oocyte and their nuclei (µm^3^) in different types of follicles, in different groups of mice 5wk after treatment with testosterone enanthate

**Groups**	**Primordial follicles**	**Primary follicles**	**Preantral follicles**	**Antral follicles**
Mean volume of oocyte				
Control	1753.47 ± 43.69^a^	3728.65 ± 337.84^a^	84345.39 ± 3486.56^a^	154475.61 + 3423.81^a^
PCOS	1731.26 ± 31.17^a^	3693.92 ± 314.73^a^	73457.70 ± 2301.40^b^	144549.03 ± 2386.15^b^
p-value	0.35	0.90	0.011	0.015
Mean volume of oocyte nucleus				
Control	518.23 ± 31.05^a^	718.04 ± 53.41^a^	2654.64 ± 195.97^a^	6677.86 ± 158.57 ^a^
PCOS	512.56 ± 19.77^a^	724.23 ± 36.19^a^	2603.21 ± 159.70^a^	6438.58 ± 169.95^a^
p-value	0.79	0.72	0.67	0.19

Values are means ± SD. a and b letters in each column indicate a significant difference between the groups (Student’s *t* test)

PCOS: Polycystic ovary syndrome

**Table IV:** Comparison of the mean thickness of zona pellucida (µm) in pre-antral and antral follicles, in different groups of mice 5 wk after treatment with testosterone enanthate

**Groups**	**Preantral follicles**	**Antral follicles**
Control	11.91 ± 0.39^a^	17.01 ± 0.42^a^
PCOS	10.59 ± 0.41^b^	15.22 ± 0.55^b^
p-value	0.016	0.011

Values are means ± SD. a and b letters in each column indicate a significant difference between the groups (Student’s *t *test).

PCOS: Polycystic ovary syndrome

**Table V:** Comparison of the mean level of testosterone, LH and FSH in different groups of mice 5 wk after treatment with testosterone enanthate

**Groups**	**Testosterone (ng/ml)**	**FSH (mIU/ml)**	**LH (mIU/ml)**
Control	3.31 ± 0.47^a^	4.07 ± 0.26^a^	2.48 ± 0.34^a^
PCOS	11.78 ± 1.21^b^	2.11 ± 0.20^b^	5.49 ± 0.67^b^
p-value	0.001	0.001	0.002

Values are means ± SD. a and b letters in each column indicate a significant difference between the groups (Student’s *t* test)

FSH: Follicle-stimulating hormone

LH: Luteinizing hormone

PCOS: Polycystic ovary syndrome

**Table VI.** Comparison of the oxidative stress and inflammatory markers in different groups of mice 5 wk after treatment with testosterone enanthate

**Group**	**MDA (µM)**	**FRAP (µmol Fe2+ /L)**	**TNF-α (pg/ml)**	**IL-6 (pg/ml)**
Control	2.81 ± 0.26 ^a^	0.94 ± 0.21 ^a^	64 ± 10.78 ^a^	289 ± 43.54 ^a^
PCOS	6.43 ± 0.40^b^	0.46 ± 0.18 ^b^	753 ± 59.57 ^b^	1382 ± 78.65 ^b^
p-value	0.001	0.020	0.001	0.001

Values are means ± SD.

MDA: Malondialdehyde

FRAP: Ferric reducing antioxidant power

TNF-α: Tumor necrosis factor-α

IL-6: Interleukin 6

PCOS: Polycystic ovary syndrome

a and b letters in each column indicate a significant difference between the groups (Student’s *t* test and Mann–Whitney U-test).

## Discussion

In this study, injections of testosterone enanthate in immature female mice induced reproductive and endocrine characteristics of PCOS such as anovulation, hyperandrogenemia, LH hypersecretion and multiple cysts. Our results revealed that ovaries in mice treated with testosterone were persisted in the diestrus phase and manifested follicular cysts with diminished granulosa cell compartment and the absence of corpora lutea which is consistent with the previous studies (23, 24). We also showed that the total volume of the ovary and the volume of the cortex significantly reduced in the PCOS group compared to the control mice which is in accordance with the results obtained by Ikeda and colleagues, who showed that treatment of immature female mice with dehydroepiandrosterone for a period of 30 days cause ovary volume reduction (25). This could be due to ovary atrophy, increase in the number of atretic follicles, reduction in the number of antral follicles and the absence of corpus luteum which occurs in the PCOS mice (25). Moreover, hyperandrogenism can induce oxidative stress, follicles atresia and ovary apoptosis which may result in a reduction in the ovary volume (26). Hyperandrogenism also accelerates early follicular growth leading to the excess of primary and preantral follicles (27), which is in agreement with the results obtained by this study. In addition, we demonstrated that the mean volume of the oocyte in preantral and antral follicles decreased significantly in the PCOS group compared to the control which is inconsistent with the results reported by Noorafshan and colleagues (28). This could be due to the changes in the follicular fluid environment such as increased levels of androgens, triglycerides and insulin which may impact the oocyte volume (29). Our results also revealed that the mean thickness of zona pellucida significantly reduced in the PCOS group when compared to the control which is confirmed by the study conducted by Ouladsahebmadarek and Khaki who indicated a decrease in the thickness of zona pellucida and the distribution of microvilli in rats following estradiol valerate injection (30). Since oocyte and follicular cells are involved in the production of zona pellucid therefore the apoptosis of granulosa cells and follicular atresia could be the underlying cause for the reduction in ZP thickness (31). 

Increased LH concentrations is another common feature of PCOS which is mainly due to the excess levels of androgen acting on the hypothalamic-pituitary axis, leading to an impaired negative feedback on LH secretion (32). We too showed a rise in the level of LH in the PCOS group which is due to the predominate increase of testosterone as a result of persistent testosterone injections (32). Although this finding is in accordance with previous studies (24, 33), it is contrary to the study of Skrtic *et al* (15). This could be due to the difference in dose or time of treatment with testosterone.

In PCOS, markers of inflammation and oxidative stress are highly correlated with the circulating androgens, as reported by González who indicated that inflammation directly stimulates the production of androgens leading to ovarian dysfunction and metabolic aberrations (34). Spaczynski and colleagues, also demonstrated that TNF-α causes proliferation and differentiation of the theca cells and consequently androgen production and hyperandrogenemia (35). Our results also showed a significant increase in the markers of inflammation such as IL-6 and TNF-α in the PCOS group compared to the control ones. Thus, our findings confirm the fact that PCOS is a pro-inflammatory state (7, 34, 36, 37) and that increasing levels of testosterone leads to an increase in inflammation.

## Conclusion

Our present study demonstrated that induction of PCOS in mice model by testosterone enanthate led to the manifestation of human PCOS such as histological changes in the ovary tissue and increase in inflammatory and oxidative stress markers. These results can be further used to study other features of PCOS and also to investigate new therapeutic approaches to PCOS.

## Note

***This article extracted from Ph.D. Thesis. (Zahra Kalhori)***
